# Genome-Wide Analysis of Soybean HD-Zip Gene Family and Expression Profiling under Salinity and Drought Treatments

**DOI:** 10.1371/journal.pone.0087156

**Published:** 2014-02-03

**Authors:** Xue Chen, Zhu Chen, Hualin Zhao, Yang Zhao, Beijiu Cheng, Yan Xiang

**Affiliations:** 1 Laboratory of Modern Biotechnology, School of Forestry and Landscape Architecture, Anhui Agricultural University, Hefei, China; 2 Key Laboratory of Crop Biology of Anhui Agriculture University, Hefei, China; International Rice Research Institute, Philippines

## Abstract

**Background:**

Homeodomain-leucine zipper (HD-Zip) proteins, a group of homeobox transcription factors, participate in various aspects of normal plant growth and developmental processes as well as environmental responses. To date, no overall analysis or expression profiling of the HD-Zip gene family in soybean (*Glycine max*) has been reported.

**Methods and Findings:**

An investigation of the soybean genome revealed 88 putative HD-Zip genes. These genes were classified into four subfamilies, I to IV, based on phylogenetic analysis. In each subfamily, the constituent parts of gene structure and motif were relatively conserved. A total of 87 out of 88 genes were distributed unequally on 20 chromosomes with 36 segmental duplication events, indicating that segmental duplication is important for the expansion of the HD-Zip family. Analysis of the Ka/Ks ratios showed that the duplicated genes of the HD-Zip family basically underwent purifying selection with restrictive functional divergence after the duplication events. Analysis of expression profiles showed that 80 genes differentially expressed across 14 tissues, and 59 HD-Zip genes are differentially expressed under salinity and drought stress, with 20 paralogous pairs showing nearly identical expression patterns and three paralogous pairs diversifying significantly under drought stress. Quantitative real-time RT-PCR (qRT-PCR) analysis of six paralogous pairs of 12 selected soybean HD-Zip genes under both drought and salinity stress confirmed their stress-inducible expression patterns.

**Conclusions:**

This study presents a thorough overview of the soybean HD-Zip gene family and provides a new perspective on the evolution of this gene family. The results indicate that HD-Zip family genes may be involved in many plant responses to stress conditions. Additionally, this study provides a solid foundation for uncovering the biological roles of HD-Zip genes in soybean growth and development.

## Introduction

Transcription factors (TFs) are types of proteins that affect many biological processes such as growth, development and cell division and respond to environmental stimuli and stressors in cells or organisms [1]. TFs bind to DNA and either activate or repress gene expression at the level of mRNA transcription [2]. Typical TFs mainly contain a DNA binding domain and a transcriptional activation domain; the former recognizes target DNA sequences while the latter initiates transcription [3]. Homeobox (HB), one of the key transcription factors families, was first identified in a set of *Drosophila* genes controlling development [4]. Each HB gene encodes a conserved 61 amino acid sequence known as the homeodomain (HD) [5], [6], which is responsible for sequence-specific DNA binding. Subsequently, HDs have also been discovered in invertebrates and vertebrates, plants and fungi [7]. In plants, *KNOTTED1*, which was isolated from maize (*Zea mays L.*), was the first HD-containing protein [8]. Since the isolation of this protein, more and more plant HD-containing genes have been isolated. According to the sequence differences and location of their HD domains, homology of the flanking sequences and other correlative domains, HD-containing proteins were classified into six families, including HD-Zip (homeodomain-leucine-zipper), KNOX (KNOTTED1-like homeobox), PHD-Finger (homeodomain-finger), Bell (bell domain), WOX (Wuschel-related homeobox) and ZF-HD (zinc finger-homeodomain) [9], [10].

Among these families, HD-Zip proteins are ubiquitous in plants and carry out essential roles in various processes of plant growth and development [11]. HD-Zip proteins contain a leucine motif adjacent to the N-terminus of the homeodomain [4], [12], [13]. The *Arabidopsis* (*Arabidopsis thaliana*) genome encompasses 48 genes believed to encode HD-Zips, which are clustered into four subfamilies based on their additional conserved domains, structures and physiological functions [14]. Members of group I and II recognize similar pseudopalindromic binding sites (BSs; CAATNATTG) [15]–[18], while HD-Zip III and IV proteins interact with slightly different sequences (GTAAT[G/C]ATTAC and TAAATG[C/T]A, respectively) [19], [20]. Members of the HD-Zip gene family contain a special conserved HD and a common conserved LZ domain [21]. The difference between the four subfamilies mainly arose in the region downstream of the LZ domain, which contains different domains. Encoded proteins of the HD-Zip II subfamily can be distinguished from HD-Zip I proteins by the presence of a conserved “CPSCE” motif, named after the five conserved amino acids Cys, Pro, Ser, Cys, Glu (in one letter code), which is located next to the LZ domain and near the C-terminus [22]. Both the HD-Zip III and HD-Zip IV subfamily proteins can be distinguished from other subfamily proteins by the presence of a StAR (steroidogenic acute regulatory protein)-related lipid-transfer (START) domain followed by an HD-START-associated domain (HD-SAD) [23], [24]. However, the HD-Zip III subfamily proteins contain an additional C-terminal MEKHLA domain, while HD-Zip IV subfamily proteins lack this motif [25].

Many members of the HD-Zip protein family have been found in a wide variety of plant species, and many efforts have been undertaken to elucidate the functions of HD-Zip genes. HD-Zip I proteins are mainly involved in responses to abiotic stress, auxin, de-etiolation, blue light signaling and the regulation of organ growth and developmental process [11]. For example, *ATHB7* and *ATHB12* from *Arabidopsis* subfamily I, which are both strongly induced by abscisic acid (ABA) and water-deficit, function as negative primary regulators of the ABA response mechanism in *Arabidopsis* [26]. Transcription of *HB1* from *Medicago truncatula* subfamily I, which is strongly induced by salt stress in root apices, regulates root architecture and lateral root emergence [27]. HD-Zip II proteins are involved in responses to illumination conditions, shade avoidance and auxin signaling [28]–[32]. *ATHB2* is the first gene that is specifically and reversibly regulated by changes in the R/FR ratio in green plants, which induces the shade avoidance response in most angiosperms [33]. *HAT2*, another member of this subfamily, was identified as an auxin-inducible gene in seedlings through DNA microarray screening [30]. *HAHB10*, a sunflower HD-ZIP II gene, participates in the induction of flowering and in the regulate of phytohormone-mediated responses to biotic stress [34].

HD-Zip III proteins control apical meristem development, embryogenesis, leaf polarity, lateral organ initiation and vascular bundle development [35]–[37]. *ATHB8* and *ATHB15* are thought to direct vascular development [38], [39]. Several studies have elucidated the mode of action of *REV*, *PHB* and *PHV*, along with *KANADI*, in controlling abaxial-adaxial patterning of lateral organs [40]. *PopREVOLUTA*(*PRE*), a *populus* class III HD-ZIP gene demonstrates in regulating the development of cambia and secondary vascular tissues [41]. HD-Zip IV proteins play crucial roles in anthocyanin accumulation, epidermal cell differentiation, trichome formation, root development and cuticle development [11], [42]. The *gl2* (*GLABRA2*) mutant shows unusual trichome expansion and ectopic root hair differentiation [13]. Recent studies have demonstrated that upregulating the expression of *HDG11*, one of the HD-Zip IV genes, allows this gene to perform novel functions in drought tolerance. This finding may help reveal how drought tolerance has evolved, as altering the expression pattern of *HDG11* may be a way in which drought tolerance evolves in nature [43]. *OCL1* (OUTER CELL LAYER1) encodes a maize HD-ZIP class IV transcription, ectopic expression of *OCL1* leads to pleiotropic phenotypic aberrations in transgenic maize plants, the most conspicuous one being a strong delay in flowering time [44].

Soybean (*Glycine max*) is one of the most economically and nutritionally crucial crops. It provides not only vegetable protein and edible oil but also essential amino acids for humans and animals. However, soybean production is threatened by drought, salinity and other environmental stresses. For example, drought reduces the yield of soybean by about 40%, affecting all stages of plant development from germination to flowering and reducing the quality of the seeds [45]. Salinity inhibit soybean growth and production and together with drought cause osmotic stress in plant [46], [47]. Thus, there is a great need to study the soybean in order to improve sustenance and better yield. To date, the soybean genome has been sequenced, which has enabled gene prediction tools and annotation to become publicly available [48]. A series of transcription factors have been studied in soybean, such as ERF, WRKY, BURP, MADS-box, MYB, NAC and so on [49]–[54]. HD-Zip family genes have been characterized in *Arabidopsis*, rice, *Populus*, maize and other species [55], [56], but no genome-wide characterization of the HD-Zip family has been performed in soybean to date. In the current study, 88 putative genes of the HD-Zip family were identified. After examining the publicly reported expression patterns of the paralogous pairs in soybean under stress, we investigated the transcript levels of six paralogous pairs under stress treatment. The results presented in this study show that the expression of the 12 soybean HD-Zip genes is stress-responsive. Our findings lay the foundation for further investigations into the biological and molecular functions of HD-Zip transcription factors in soybean.

## Results

### Identification of HD-Zip gene family in soybean

The HD-Zip genes, characterized by the existence of HD and LZ domains, have previously been systematically analyzed in *Arabidopsis*, rice, *Populus* and maize. In the present study, to gain insight into the HD-Zip gene family in soybean, we first used the HD-Zip genes of *Arabidopsis* to perform a BLASTP search against the soybean genome database (release 1.0). According to the features of each HD-Zip subfamily, four representatives were randomly chosen as secondary queries from the resulting sequences. Using this method, a total of 100 putative HD-Zip genes were obtained. SMART and Pfam analysis were performed to retain those putative genes that included both HD and LZ domains. This analysis revealed 88 members in soybean, which is greater than that identified in other representative species, including *Arabidopsis* (48), rice (48), maize (55) and *Populus* (63) [55], [56]. The 88 identified soybean HD-Zip genes in our study were designated *Gmhdz1* to *Gmhdz88* following the nomenclature proposed in a previous study [55]. The encoded proteins varied from 206 to 853 amino acids (aa) in length, with an average of 462 aa, which is similar to that reported in *Populus* (465 aa) [57]. The details about other parameters of the nucleic acid and protein sequences are provided in Table 1.

**Table 1:** List of 88 HD-Zip genes identified in soybean and their sequence characteristics (bp, base pair; aa, amino acids; D, Dalton).

		Arabidopsis	Protein			Chr.	ORF length	Exons
Name	Sequenced ID	orthologs locus	Length(a.a.)	Mol.Wt.(Da)	PI		(bp)	
Gmhdz1	Glyma0041s0035	HAT14	309	34425.2	6.33	scaffold_41	930	3
Gmhdz2	Glyma01g04890	ATHB1	345	39174	4.63	1	1038	3
Gmhdz3	Glyma01g05230	ATHB13	283	32185.9	6.08	1	852	3
Gmhdz4	Glyma01g38390	ATHB21	214	25074.2	6.26	1	645	3
Gmhdz5	Glyma01g40450	HAT22	283	31863.1	8.5	1	852	3
Gmhdz6	Glyma01g41581	ATHB2	268	30100.9	8.16	1	807	4
Gmhdz7	Glyma01g45070	PDF2	731	80339.9	5.92	1	2211	10
Gmhdz8	Glyma02g02290	ATHB13	295	33680.6	6.19	2	888	3
Gmhdz9	Glyma02g02630	ATHB1	345	39145.3	4.67	2	1038	3
Gmhdz10	Glyma02g06560	ATHB21	212	25015.1	5.82	2	639	3
Gmhdz11	Glyma02g28860	HAT14	309	34206.3	8.44	2	990	4
Gmhdz12	Glyma03g01860	ANL2	835	91244.1	5.97	3	2508	9
Gmhdz13	Glyma03g26701	HAT3	310	34415.1	6.41	3	933	4
Gmhdz14	Glyma03g30200	HAT14	280	30983.9	8.03	3	867	4
Gmhdz15	Glyma03g34710	ATHB51	233	27315.9	6.03	3	702	3
Gmhdz16	Glyma04g05200	HAT14	247	28321.2	9.32	4	873	3
Gmhdz17	Glyma04g09000	CNA1	844	92741	6.03	4	2535	18
Gmhdz18	Glyma04g34341	ATHB1	289	32133.2	4.64	4	870	4
Gmhdz19	Glyma04g40960	ATHB7	245	28360.5	5.44	4	738	2
Gmhdz20	Glyma05g01390	ATHB1	331	37413.2	4.77	5	996	3
Gmhdz21	Glyma05g04990	ATHB2	298	33329.2	7.68	5	897	4
Gmhdz22	Glyma05g23150	HAT22	305	33662.1	8.67	5	918	3
Gmhdz23	Glyma05g30000	PHB	853	93958.1	6.06	5	2562	18
Gmhdz24	Glyma05g30940	ATHB16	345	38838.9	4.89	5	1038	4
Gmhdz25	Glyma05g33520	HDG11	713	79304.9	6.37	5	2142	10
Gmhdz26	Glyma06g09100	CNA1	845	92923.2	6.03	6	2538	18
Gmhdz27	Glyma06g13890	ATHB7	251	29036.2	5.53	6	756	2
Gmhdz28	Glyma06g20230	ATHB1	326	36832.4	4.71	6	981	3
Gmhdz29	Glyma07g01940	CNA1	838	92450.7	6.06	7	2517	18
Gmhdz30	Glyma07g01950	CNA1	841	92947.1	6.12	7	2526	11
Gmhdz31	Glyma07g02220	GL2	751	84101	5.65	7	2256	11
Gmhdz32	Glyma07g05800	ATHB12	238	27335.2	5.11	7	717	2
Gmhdz33	Glyma07g08340	ANL2	829	90529.4	5.97	7	2490	9
Gmhdz34	Glyma07g14270	HAT3	308	34006.9	7.66	7	954	5
Gmhdz35	Glyma07g34230	ATHB17	206	23270.4	8.62	7	642	4
Gmhdz36	Glyma08g06190	HDG11	721	80097.8	6.21	8	2166	10
Gmhdz37	Glyma08g13110	PHB	833	91561.5	6.15	8	2550	18
Gmhdz38	Glyma08g14130	ATHB16	312	35437	4.93	8	939	3
Gmhdz39	Glyma08g15771	HAT14	377	41903	6.1	8	1134	4
Gmhdz40	Glyma08g21610	CNA1	838	92386.5	6.06	8	2517	18
Gmhdz41	Glyma08g21620	CNA1	843	93335.6	6.17	8	2532	18
Gmhdz42	Glyma08g21890	GL2	748	83710.8	5.66	8	2247	11
Gmhdz43	Glyma08g40705	ATHB1	320	36687.7	4.79	8	963	3
Gmhdz44	Glyma08g40970	ATHB13	280	31882.6	5.68	8	843	3
Gmhdz45	Glyma09g02750	PHB	852	93475.4	5.89	9	2559	18
Gmhdz46	Glyma09g07051	HAT14	237	26645.3	8.49	9	714	3
Gmhdz47	Glyma09g16790	HAT14	327	36191.6	8.09	9	984	4
Gmhdz48	Glyma09g26600	ANL2	776	85098.6	5.53	9	2331	9
Gmhdz49	Glyma09g29810	HDG11	722	79550.4	6.25	9	2169	10
Gmhdz50	Glyma09g34061	HDG5	800	88979.7	5.69	9	2403	11
Gmhdz51	Glyma09g37410	ATHB20	270	30941.7	6.35	9	813	3
Gmhdz52	Glyma09g37680	ATHB2	229	25443.4	8.52	9	690	3
Gmhdz53	Glyma09g40130	ANL2	820	89611.68	6.01	9	2463	9
Gmhdz54	Glyma10g38280	ANL2	765	83880.5	5.77	10	2298	9
Gmhdz55	Glyma10g39720	PDF2	740	82507.11	6.25	10	2334	10
Gmhdz56	Glyma11g00570	PDF2	732	80460.95	5.86	11	2199	10
Gmhdz57	Glyma11g03850	ATHB2	285	31968	8.47	11	858	4
Gmhdz58	Glyma11g04840	HAT22	283	31683	8.78	11	852	3
Gmhdz59	Glyma11g20520	REV	842	92062.9	5.75	11	2529	18
Gmhdz60	Glyma11g37920	ATHB5	314	35762.3	4.87	11	945	3
Gmhdz61	Glyma12g08080	REV	841	92012.8	5.75	12	2526	18
Gmhdz62	Glyma12g10710	HDG2	727	79323.7	5.56	12	2286	11
Gmhdz63	Glyma12g32050	HDG2	781	84642.9	5.74	12	2346	11
Gmhdz64	Glyma13g00310	HAT3	213	24498.9	8.92	13	642	3
Gmhdz65	Glyma13g05270	ATHB20	291	33146.1	7.25	13	876	3
Gmhdz66	Glyma13g23890	ATHB1	285	32676.2	4.74	13	858	4
Gmhdz67	Glyma14g10370	HAT14	305	34410.3	6.23	14	918	3
Gmhdz68	Glyma15g01960	GL2	751	83589.59	5.84	15	2256	11
Gmhdz69	Glyma15g13640	PHB	846	92842	5.89	15	2541	18
Gmhdz70	Glyma15g18320	HAT22	226	25323.8	6.95	15	681	3
Gmhdz71	Glyma15g42380	HAT14	384	42520.6	5.63	15	1155	4
Gmhdz72	Glyma16g02390	ATHB12	245	28334.2	5.14	16	738	2
Gmhdz73	Glyma16g34350	HDG11	718	79148.95	6.3	16	2157	10
Gmhdz74	Glyma17g06380	HAT14	209	23720.1	9.27	17	630	3
Gmhdz75	Glyma17g10490	ATHB1	329	37381.1	4.82	17	990	3
Gmhdz76	Glyma17g15380	ATHB2	299	33672.6	7.67	17	900	4
Gmhdz77	Glyma17g16930	HAT22	312	34422.8	8.05	17	939	3
Gmhdz78	Glyma18g01830	ATHB5	322	36683.3	4.82	18	969	3
Gmhdz79	Glyma18g15970	ATHB13	279	31693.4	5.67	18	840	3
Gmhdz80	Glyma18g16390	ATHB1	264	30554.6	4.71	18	975	3
Gmhdz81	Glyma18g45970	ANL2	773	84777.39	6.14	18	2469	9
Gmhdz82	Glyma18g48880	HAT3	289	31924.6	6.46	18	870	4
Gmhdz83	Glyma18g49290	ATHB20	268	30527.3	7.17	18	807	3
Gmhdz84	Glyma19g01300	ATHB1	284	32387.9	4.71	19	855	4
Gmhdz85	Glyma19g02490	ATHB20	292	33500.3	6.9	19	879	3
Gmhdz86	Glyma19g33100	HAT14	270	30215.3	8.51	19	879	4
Gmhdz87	Glyma19g37380	ATHB51	214	25143.3	7.76	19	645	3
Gmhdz88	Glyma20g01770	ATHB17	218	24720.2	9.13	20	660	4

### Phylogenetic and structural analyses of the HD-Zip proteins in soybean

**Figure 1:**
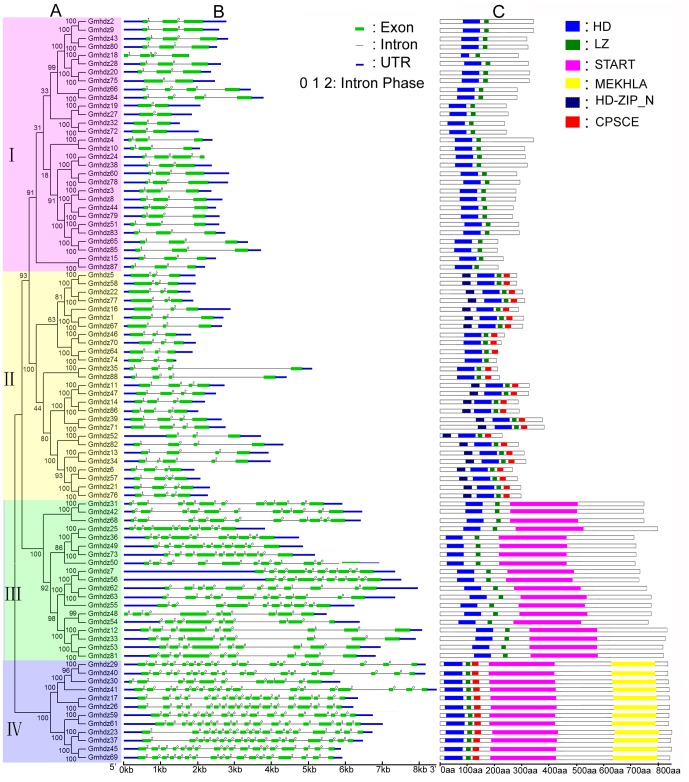
Phylogenetic relationships, gene structure and motif compositions of soybean HD-Zip genes. A. The unrooted tree was generated with the MEGA5.0 program using the full-length amino acid sequences of the 88 soybean HD-Zip proteins by the Neighbor-Joining (NJ) method, with 1,000 bootstrap replicates. The percentage bootstrap scores higher than 50% are indicated on the nodes. The tree shows four major phylogenetic subfamilies (subfamily I to IV) indicated with different colored backgrounds. B. Exon/intron organization of soybean HD-Zip genes. Green boxes represent exons and black lines represent introns. The untranslated regions (UTRs) are indicated by blue boxes. The numbers 0, 1 and 2 represents the splicing phases. The sizes of exons and introns can be estimated using the scale at the bottom. C. Schematic representation of the conserved motifs in soybean HD-Zip proteins elucidated from publicly available data. Each colored box represents a motif in the protein, with the motif name indicated in the box on the right. The length of the protein and motif can be estimated using the scale at the bottom.

To evaluate the evolutionary relationships among soybean HD-Zip proteins, an unrooted phylogenetic tree of the 88 soybean protein sequences was generated, with 1,000 bootstrap replicates. The soybean HD-Zip family was further divided into four major subfamilies (I to IV) with >50% bootstrap values (Figure 1A). Subfamily III has the fewest HD-Zip gene members (12), while subfamily I contains the most members (30), followed by subfamily II (27) and IV (19). This distribution is similar to that observed for HD-Zip genes in other species. Based on phylogenetic analysis, we identified 41 sister pairs (Table 2), all of which had high bootstrap support (>94%).

**Table 2:** Divergence between HD-Zip genes pairs in soybean.

Paralogous pairs	Ks	Ka	Ka/Ks	Duplication Date(MY)	Duplicate type
Gmhdz2-Gmhdz9	0.101	0.028	0.275	8.25	Segmental
Gmhdz3-Gmhdz8	0.089	0.111	0.214	7.3	Segmental
Gmhdz5-Gmhdz58	0.139	0.038	0.274	11.37	Segmental
Gmhdz6-Gmhdz57	0.201	0.027	0.136	16.44	Segmental
Gmhdz7-Gmhdz56	0.109	0.021	0.189	8.96	Segmental
Gmhdz11-Gmhdz47	0.224	0.043	0.19	18.39	Segmental
Gmhdz12-Gmhdz33	0.142	0.015	0.109	11.6	Segmental
Gmhdz13-Gmhdz34	0.145	0.044	0.3	11.9	Segmental
Gmhdz14-Gmhdz86	0.154	0.068	0.442	12.63	Segmental
Gmhdz15-Gmhdz87	0.127	0.043	0.336	10.44	Segmental
Gmhdz17-Gmhdz26	0.115	0.006	0.054	9.4	Segmental
Gmhdz18-Gmhdz28	0.181	0.047	0.257	14.87	Segmental
Gmhdz19-Gmhdz27	0.117	0.021	0.182	9.62	Segmental
Gmhdz20-Gmhdz75	0.156	0.061	0.388	12.81	Segmental
Gmhdz21-Gmhdz76	0.13	0.038	0.292	10.61	Segmental
Gmhdz22-Gmhdz77	0.142	0.047	0.334	11.64	Segmental
Gmhdz23-Gmhdz37	0.084	0.017	0.205	6.85	Segmental
Gmhdz24-Gmhdz38	0.145	0.038	0.265	11.88	Segmental
Gmhdz25-Gmhdz36	0.098	0.025	0.256	8.02	Segmental
Gmhdz31-Gmhdz42	0.118	0.042	0.353	9.63	Segmental
Gmhdz32-Gmhdz72	0.074	0.034	0.461	6.03	Segmental
Gmhdz35-Gmhdz88	0.132	0.02	0.149	10.78	Segmental
Gmhdz39-Gmhdz71	0.207	0.036	0.173	16.97	Segmental
Gmhdz43-Gmhdz80	0.218	0.064	0.295	17.83	Segmental
Gmhdz44-Gmhdz79	0.106	0.011	0.102	8.68	Segmental
Gmhdz45-Gmhdz69	0.188	0.013	0.069	15.37	Segmental
Gmhdz46-Gmhdz70	0.114	0.024	0.214	9.35	Segmental
Gmhdz49-Gmhdz73	0.093	0.009	0.096	7.64	Segmental
Gmhdz51-Gmhdz83	0.112	0.058	0.518	9.2	Segmental
Gmhdz52-Gmhdz82	0.134	0.038	0.287	10.97	Segmental
Gmhdz59-Gmhdz61	0.115	0.007	0.062	9.44	Segmental
Gmhdz60-Gmhdz78	0.163	0.033	0.199	13.39	Segmental
Gmhdz64-Gmhdz74	0.131	0.055	0.42	10.74	Segmental
Gmhdz65-Gmhdz85	0.205	0.046	0.223	16.83	Segmental
Gmhdz53-Gmhdz81	0.14	0.017	0.12	11.47	Segmental
Gmhdz66-Gmhdz84	0.093	0.024	0.257	7.66	Segmental
Gmhdz29-Gmhdz40	0.088	0.005	0.062	7.18	Segmental
Gmhdz40-Gmhdz41	0.1092	0.0226	0.2065	8.95	Tandem
Gmhdz29-Gmhdz30	0.112	0.0138	0.1231	9.18	Tandem

To gain further insights into the structural diversity of the HD-Zip genes, we compared the exon/intron organization in the coding sequences of individual HD-Zip genes in soybean (Figure 1B). Most closely related members in the same subfamilies share similar exon/intron structures and intron numbers, which was consistent with the characteristics defined in the above phylogenetic analysis. For instance, the HD-Zip genes in subfamily I and II contain two to five exons, while those in subfamily IV contain 9 to 11 exons and those in subfamily III possess 18 exons, with the exception of *Gmhdz30*, which harbors 11 exons.

To obtain intron gain/loss information for all sister pairs, we also compared the intron/exon structures of the genes that clustered together at the terminal branch of the phylogenetic tree. Among these, five pairs showed changes in their intron/exon structure, including *Gmhdz18/-28*, *Gmhdz20/-75*, *Gmhdz24-/38*, *Gmhdz52/-82* and *Gmhdz13/-34* (Fig. 1B), which only occurred in subfamily I and II. Through comparison of the five pairs with neighbouring genes, we found that *Gmhdz75* and *Gmhdz82* lost one intron during the long evolutionary period, while *Gmhdz24*, *Gmhdz18* and *Gmhdz34* gained one intron.

A total of 88 HD-Zip genes from soybean were subjected to analysis with MEME to reveal conserved motifs shared among related proteins. Thirty conserved motifs were identified (Fig. 1C); the details are shown in Figure S1 and Table S1. Each of the putative motifs obtained from MEME was annotated by searching Pfam and SMART. To simplify the MEME results, if two or more motifs among the 30 motifs identified represented the same domain and stayed close, they were merged and displayed as a domain district, while the motifs that were not annotated were not shown in Figure 1C. After this process was completed, it became clear that most of the closely related members had common motif compositions, suggesting functional similarities among HD-Zip proteins within the same subfamily.

Among these, the conserved motifs encoding the HD and LZ domain were found in all soybean HD-Zip genes; these were the most conserved motifs that were identified. The CPSCE motif was found in the majority of members of the HD-Zip III and HD-Zip II subfamilies with the exception of *Gmhdz74*. HD-Zip_N is less conserved within the HD-Zip II subfamily, as it was not found in *Gmhdz35*, *-46*, *-64*, *-70*, *-74* or *-88*. The MEKHLA domain, which is specific to subfamily III, was found only in subfamily III proteins (12 members). Motifs representing the START region were identified in subfamily III and IV genes.

### Chromosomal location and gene duplication

**Figure 2:**
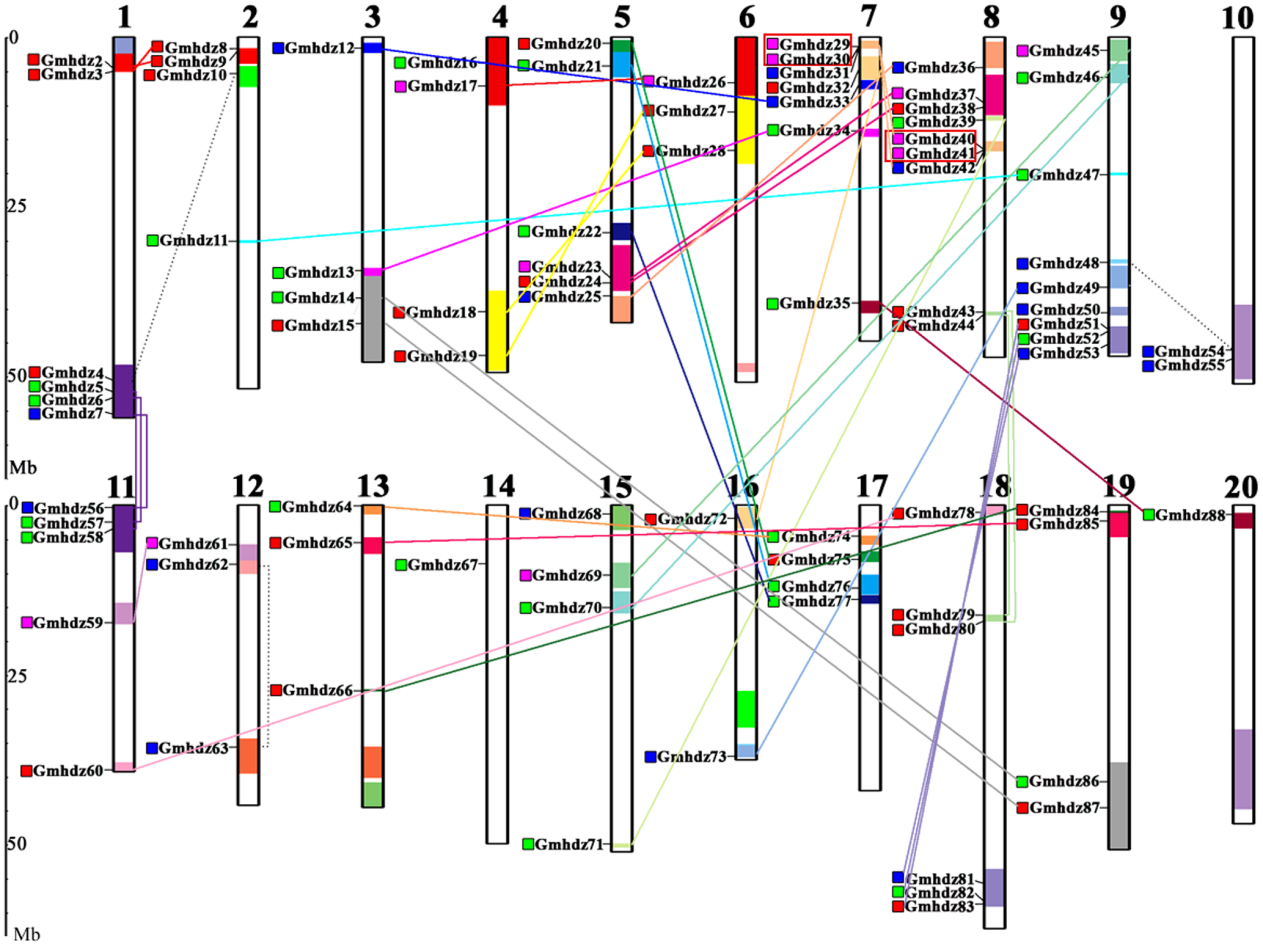
Chromosomal locations of soybean HD-Zip genes. Colored boxes ahead of the gene names represent the classes to which each HD-Zip gene belongs (HD-Zip I, red; HD-Zip II, green; HD-Zip III, pink; HD-Zip IV, blue). The 88 HD-Zip genes were mapped to the 20 chromosomes, while only one gene (*Gmhdz1*) resides on unassembled scaffolds. The data used to generate the schematic diagram of the genome-wide chromosome organization were obtained from the SoyBase browser. Only segmental duplicated blocks including HD-Zip genes are indicated with the same colors. Small boxes connected by colored line (two types) indicate corresponding sister gene pairs, of which the genes connected by solid lines are located in the homologous duplicated blocks, while genes connected by the dashed line were observed in the duplicated blocks shown with different colors. Tandemly duplicated genes are indicated with red boxes. The scale represents the length of the chromosome.

A total of 87 of the 88 soybean HD-Zip genes were mapped to the 20 soybean chromosomes, while only one gene (*Gmhdz1*) was mapped to as yet unattributed scaffolds (Figure 2). The soybean HD-Zip genes are unevenly distributed among all chromosomes; chromosomes 14 and 20 contain only one HD-Zip gene, while chromosomes 8 and 9 contain nine, the maximum number HD-Zip genes per chromosome. Among the HD-Zip subfamilies, HD-Zip III and HD-Zip IV are mainly located on chromosomes 7, 8 and 9, while HD-Zip I and II are located on chromosomes 1, 2, 3, 18 and 19.

Previous analysis of the soybean genome has revealed that the paralogs within a gene family were mainly derived from genome duplications that occurred approximately 59 and 13 million years ago(Mya) [48]. In this study, we identified 37 related duplicated blocks (Table S2). Of the 87 mapped HD-Zips, only one gene (*Gmhdz67*) is located outside of a duplicated block. Moreover, 27 block pairs cover 37 HD-Zip sister pairs, and 10 duplicated blocks only harbor HD-Zips on one of the blocks and lack the corresponding duplicates, suggesting that dynamic changes may have occurred after segmental duplication, leading to the loss of some genes.

It is noteworthy that among the 87 genes, two gene pairs (*Gmhdz29/-30*, *Gmhdz40/-41*) were detected within a distance of less than 5 kb (<100 kb) on chromosomes 7 and 8, which may have resulted from tandem duplication (Figure 2). Alignment analysis of protein sequences using the Smith-Waterman algorithm (http://www.ebi.ac.uk/Tools/psa/) showed that two pairs (*Gmhdz29/-30*, *Gmhdz40/-41*) have high sequence similarities (the former is 98%, the latter is 96.3%) between two counterparts of each gene pair and therefore meet the standards of tandem duplicates, the sequence similarities of the other paralogous pairs were showed in Table S3. Analysis of HD-Zip paralogous pairs showed that 37 out of 41 gene pairs remain in conserved positions on segmental duplicated blocks, providing strong evidence that gene duplication has made an important contribution to soybean HD-Zip gene expansion (Figure 2 and Table 2). According to the ratio of nonsynonymous to synonymous substitutions (Ka/Ks), the history of selection acting on coding sequences can be measured [58]. A pair of sequences will have Ka/Ks<1 if one sequence has been under purifying selection but the other has been drifting neutrally, while Ka/Ks = 1 if both sequences are drifting neutrally and rarely, Ka/Ks>1 at specific sites that are under positive selection [59]. A summary of Ka/Ks for 39 HD-Zip duplicated pairs is shown in Table 2 were less than 0.6. This result suggests that all gene pairs have evolved mainly under the influence of purifying selection. Based on the divergence rate of 6.1×10^−9^ synonymous mutations per synonymous site per year as previously proposed for soybean [60], duplications of these 41 paralogous pairs was estimated to have occurred between 6.03 to 18.39 Mya (Table 2).

### Comparative analysis of the HD-Zip genes in soybean, *Arabidopsis* and rice

**Figure 3:**
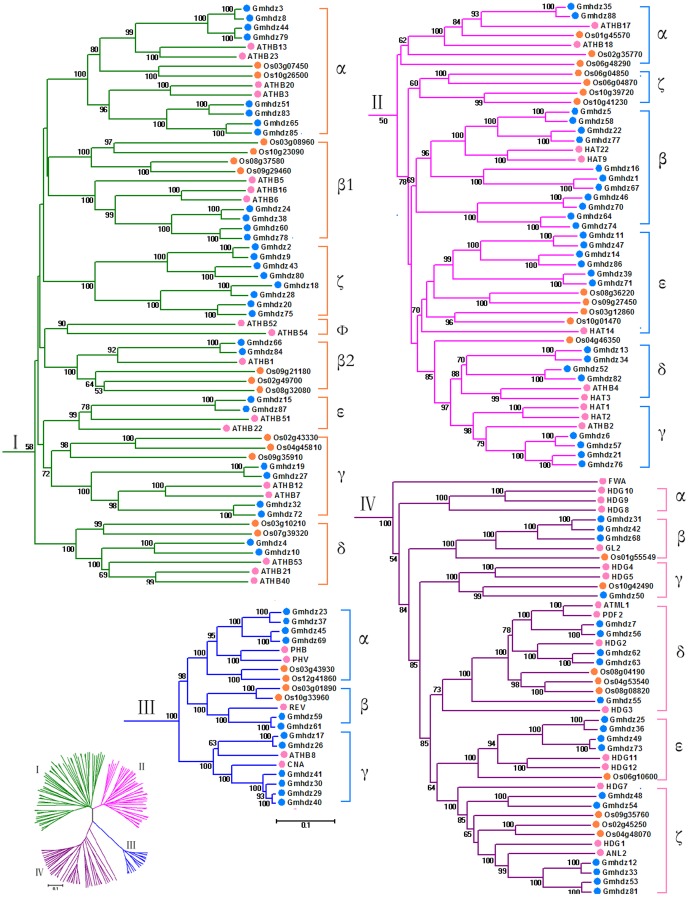
Phylogenetic trees of full-length HD-Zip domain proteins from soybean, *Arabidopsis* and rice. The tree was generated with the MEGA5.0 program using the NJ method. Numbers at nodes indicate the percentage bootstrap scores, and only bootstrap values higher than 50% from 1,000 replicates are shown. Soybean, *Populus* and *Arabidopsis* HD-Zip proteins were marked with different colored dots. The scale bar corresponds to 0.1 estimated amino acid substitutions per site. The unrooted tree in the lower-left corner was constructed with the same method.

The abundance of soybean HD-Zip genes compared to that in other plant species may have been derived from multiple gene duplication events, represented by a whole-genome duplication following multiple segmental and tandem duplications. To verify this hypothesis, we first constructed a NJ phylogenetic tree with MEGA5.0 using full-length HD-Zip protein sequence alignments of soybean, rice and *Arabidopsis* HD-Zip proteins to reveal the evolutionary relationships among plant HD-Zip proteins. At first glance, four subfamilies (I to IV) are evident in the tree (Fig. 3), the same as described previously and with significant statistical support. The phylogenetic tree reveals that the plant HD-Zip sequence distribution predominates with species bias (Fig. 3). HD-Zip I genes generally comprise the largest subfamilies in these plant species, while HD-Zip III genes comprise the smallest number of HD-Zip members.

Both subfamilies contain rice, *Arabidopsis* and soybean HD-Zip genes, suggesting that the main characteristics of this family in plants were generated before the dicot-monocot split. To clarify the paralogous and orthologous relationships among this family, we further divided the subfamilies into subclasses. We labeled the clades in the tree using previously defined clades from studies of *Arabidopsis* and rice, as shown in Figure 3. Subfamily I was divided into seven subclasses, i.e., α, β, γ, δ, ε, φ and ζ. Clade ζ contains only sequences from soybean, while clade φ exclusively contains *Arabidopsis* genes. Clade ε contains sequences from both soybean and *Arabidopsis*, while no members of rice were detected in this subclades, suggesting that rice lost its members of this group during the long period of evolution. The HD-Zip II subfamily was divided into six subclasses, from α to ζ, while α to ε were designated as described by Ciarbelli et al. (2008) [33]. Clade ζ is entirely composed of HD-Zip genes from rice. The HD-Zip III subfamily was classified into three subclades (designated α, β and γ) using the definitions of the previous studies. Clade γ excludes rice genes. The HD-Zip IV subfamily was also divided into six subclades. All of the genes in Clade α originated from *Arabidopsis*. The bootstrap values for all of the subclades were quite high, suggesting that the genes in each subclade may share a similar origin. Only one pair of orthologous genes from soybean and rice was identified, i.e., *Gmhdz50* and *Os10g42490* in subfamily IV. Most genes in the HD-Zip family are contained in paralogous pairs. This lineage-specific pattern suggests that HD-ZIP genes in these subgroups may be expanded and then diversified after the monocot-eudicot division.

### Expression profiling of soybean HD-Zip genes under drought and salt stress

**Figure 4:**
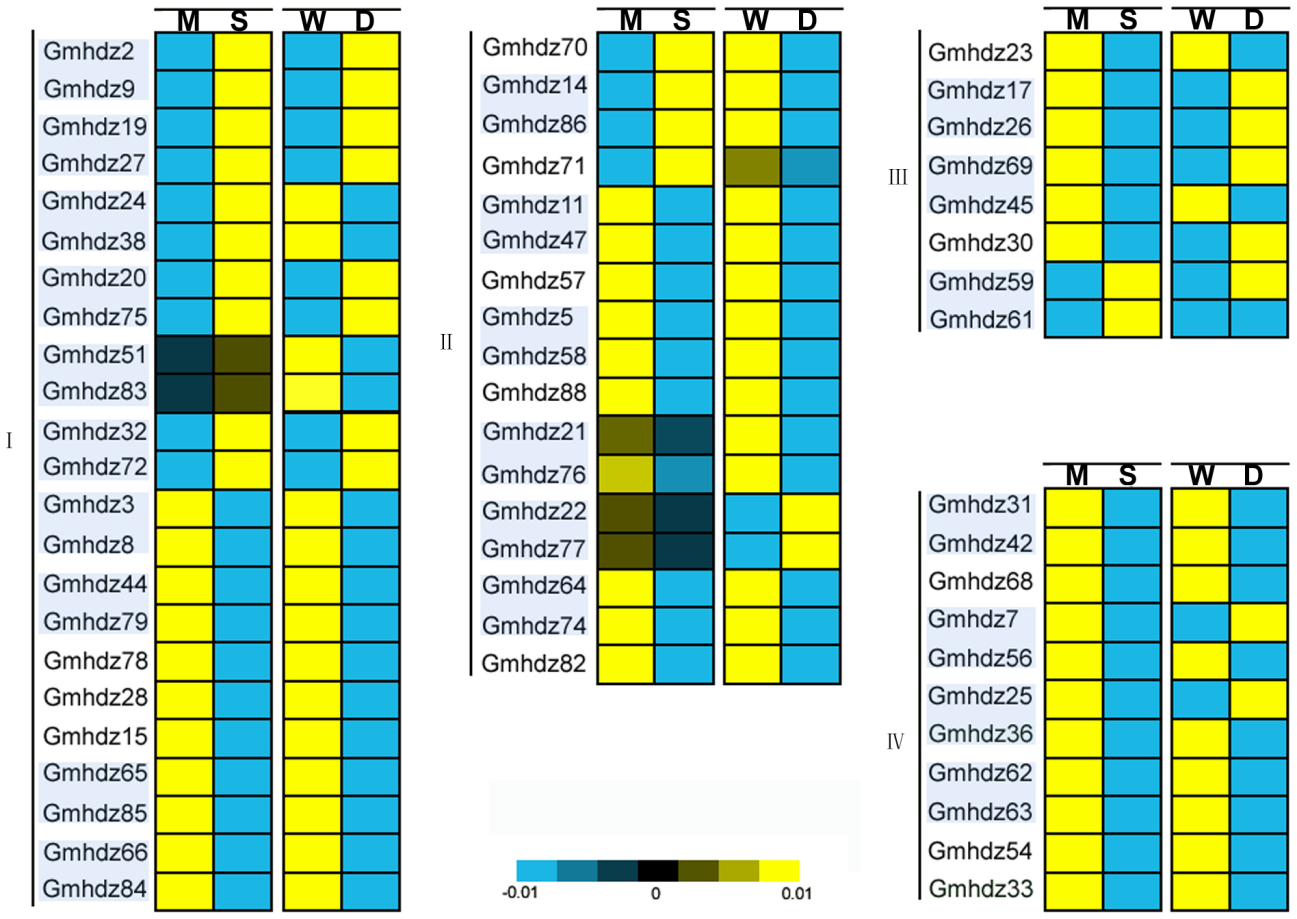
Differential expression of soybean HD-Zip genes under salinity and drought stress. Expression is indicated as fold-change of experimental treatments relative to control samples and visualized in heatmaps. Color scale represents log2 expression values, with yellow representing low levels and blue indicating high levels of transcript abundance. To increase the contrast in this figure, the color scale values were reduced. The heatmap shows hierarchical clustering of 59 genes under salinity and drought stress. The pairs with light blue background are paralogous genes. Microarray data (under accession numbers GSE40627 and GSE41125) were obtained from the NCBI GEO database. M, mock; S, salinity stress; W, well-watered; D, drought stress. The paralogous pairs are indicated with a light blue background.

To gain more insights into the roles of soybean HD-Zip genes in salinity and drought tolerance, we reanalyzed the expression profiles of all soybean HD-Zip genes in response to drought and salinity stresses using publicly available microarray data. Fifty-nine HD-Zip genes were included on both GSE40627 and GSE41125,including class I (23 genes), class II (17 genes), class III(8 genes) and class IV(11 genes) (Table S4). The expression of most HD-Zip genes was suppressed or induced under both stresses (Fig. 4). Salt stress caused upregulation of 16 genes, including 12 genes from HD-Zip I (*Gmhdz2*, -*9*, -*19*, -*20*, -*24*, -*27*, -*32*, -*38*, -*51*, -*72*, -*75* and -*83*), four gene from HD-Zip II (*Gmhdz14*, -*70*, -*71* and *-86*) and two genes in the HD-Zip III subfamilies (*Gmhdz59* and -*61*). In response to drought stress, 40 genes showed downregulation, whereas the transcripts of 18 were significantly upregulated (Fig. 4). Nine genes were upregulated under both salt and drought stress treatments, including eight genes from HD-Zip I (*Gmhdz2*, -*9*, -*19*, -*20*, -*27*, -*32*, -*72* and -*75*) and one gene from HD-Zip III (*Gmhdz59*), while 33 genes were downregulated under both stresses. The responses of HD-Zip genes to stress also differed among some genes. For instance, eight genes (*Gmhdz14*, -*24*, -*38*, *-51*, -*70*, -*71*, -*83* and -*86*) were significantly upregulated under salt stress, whereas downregulation were observed in these genes under drought stress (Fig. 4).

**Figure 5:**
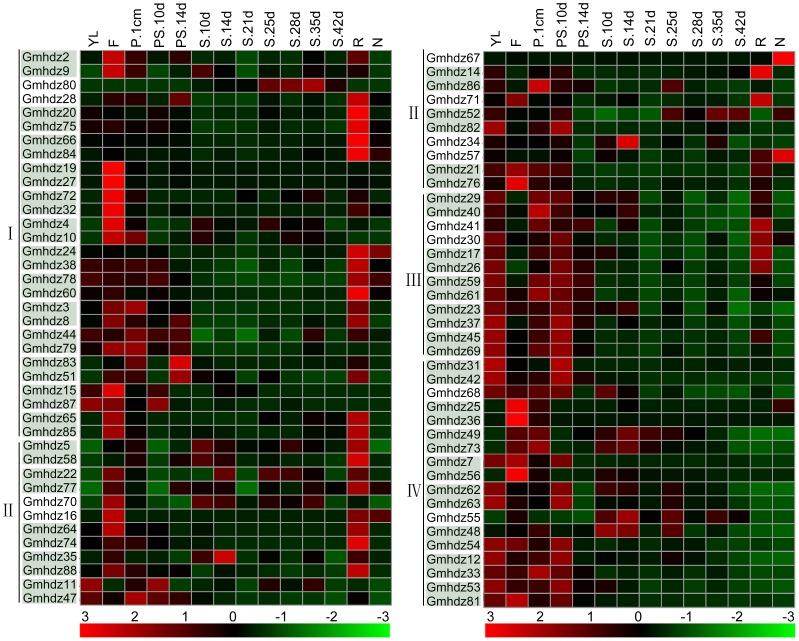
Hierarchical clustering of expression profiles of soybean HD-Zip genes in different tissues. The RNA-seq relative expression data of 14 tissues was used to re-construct expression patterns of soybean genes. Sources of the samples are as follows: YL (young leave), F (flower), P.1cm (one cm pod), PS.10d (pod shell 10DAF), PS.14d (pod shell 14DAF), S.10d (seed 10DAF), S.14d (seed 14 DAF), S.21d (seed 21DAF), S.25d (seed 25DAF), S.28d (seed 28DAF), S.35d (seed 35DAF), S.42d (seed 42DAF), R (root), N (nodule). The raw data was downloaded from the website http://soybase.org/soyseq/. Gene names in light green background showed paralogous pairs. The normal relative expressions of 88 HD-Zip genes were in the Table S6.

Duplicate genes may have different evolutionary fates, i.e., nonfunctionalization, neofunctionalization or subfunctionalization, which may be indicated by the divergence in their expression patterns. From the above results, we determined that there are 41 paralogous gene pairs in the soybean HD-Zip gene family. Based on in silico expression data from 23 paralogous pairs in response to salt and drought stress, expression divergence of drought stress was evident in three pairs(*Gmhdz69/-45, Gmhdz7/-56, Gmhdz25/-36*), which supports the notion that the expression of paralogs can diverge significantly after duplication. For example, *Gmhdz45* was upregulated in response to drought stress, whereas its duplicated counterpart *Gmhdz69* was downregulated. As shown in Figure 4, most pairs of paralogs in soybean share similar expression patterns and thus show functional redundancy. From the online database, 8 Gmhdz genes (2 for HD-Zip I, 5 for HD-Zip II and 1 for HD-Zip IV), were undetectable at the transcription level in all 14 tissues (Table S5 and Figure 5). Most soybean HD-Zip genes have a broad expression spectrum. The genes in different subfamilies had their primary abundant transcripts in different tissues, such as HD-Zip I and HD-Zip II in flower and root, both HD-Zip III and IV in young leaf, one com pod and pod shell of 10 days after flowering, some of HD-Zip IV genes also highly expressed in flower. There are a few genes strongly expressed in seed and node. From the expression data of the 34 detected paralogous pairs in14 soybean tissues, expression divergence was also obviously evidenced. For example, *Gmhdz35* from HD-Zip II highly expressed in seed of 14 days after flowering while *Gmhdz88* was undetectable. However, the paralogous pairs also have the same expression pattern, such as both *Gmhdz19* and *Gmhdz27* from HD-Zip I strongly expressed in flower and barely in other tissues.

### Examination of HD-Zip gene expression by qRT-PCR

**Figure 6:**
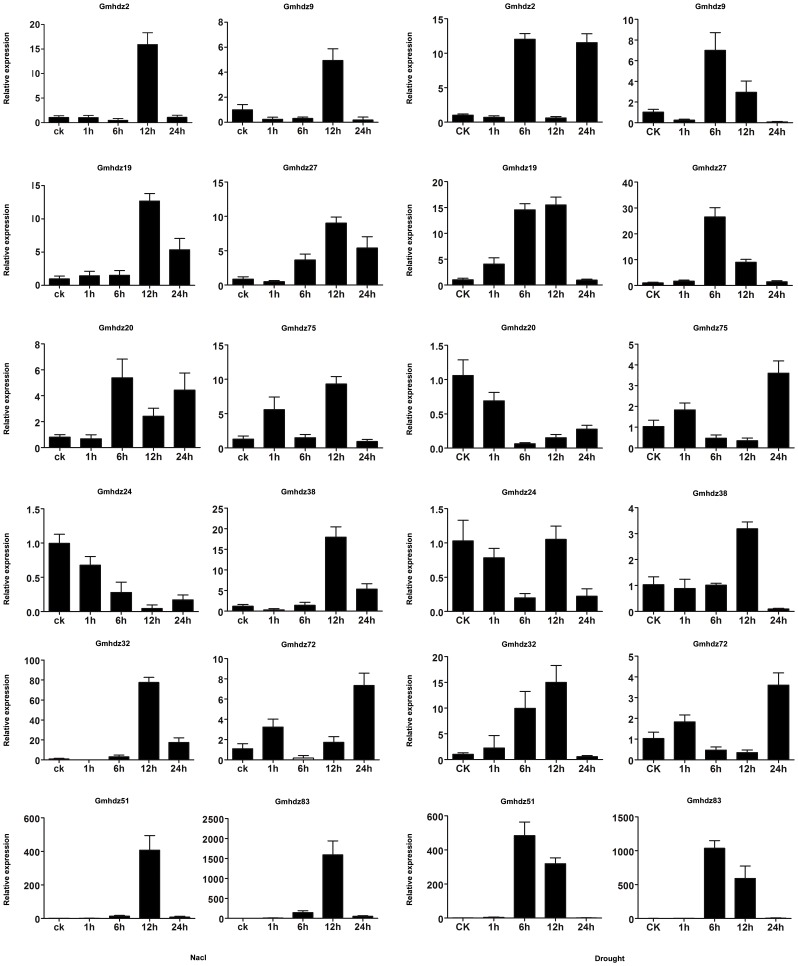
Expression analysis of 12 selected HD-Zip genes under drought and salinity stress using qRT-PCR. The relative mRNA abundance of 12 selected HD-Zip genes was normalized with respect to the reference gene *CYP2* under drought and salinity stress treatment. Bars represent standard deviations (SD) of three technical replicates. The X-axes show the time courses of stress treatments for each gene.

The HD-Zip I genes have been shown to play major roles in abiotic stress [61]. Soil salinity, one of the most severe abiotic stresses, hinders the improvement of agricultural productivity on nearly 20% of irrigated land worldwide [46]. As water becomes limited, plants redistribute this valuable resource by restricting transpiration and growth, and they frequently flower early [62]. Therefore, there is a need to identify the master regulators and their regulatory pathways involved in stress adaptation. To screen soybean HD-Zip genes regulated by salt and drought stress, qRT-PCR was employed to validate 12 candidate genes (*Gmhdz2*, -*9*, -*19*, -*20*, -*24*, -*27*, -*32*, -*38*, -*51*, -*72*, -*75* and -*83*) from HD-Zip I subfamily that are highly induced by salt stress based on microarray data. The 12 genes are also highly induced by drought stress (except for *Gmhdz24*, *-38*, *-51* and *-83*, which are downregulated). In addition, the 12 genes comprise six paralogous pairs. The qRT-PCR results showed that all 12 HD-Zip genes were drought-and salinity-responsive, but some differences were observed among these genes (Fig. 6). Under NaCl treatment, only *Gmhdz20* was highly expressed at a comparatively early stage (6 h after treatment), whereas the levels of *Gmhdz2*, -*9*, -*19*, -*27*, -*32*, -*38*, -*61*, -*75* and -*83* expression peaked at 12 h after treatment. *Gmhdz24* was downregulated by NaCl treatment across all time points. Notably, *Gmhdz51* and -*83* were strongly upregulated (>400-fold) at 12 h after NaCl treatment but were dramatically downregulated thereafter (Fig. 6). Under drought stress, the highest expression levels of *Gmhdz9*, *-27*, -*51*, -*75* and -*83* were found at 6 h after treatment, while those of *Gmhdz24*, -*32* and *-38* were observed later, at 12 h after treatment.

Notably, *Gmhdz72* was highly expressed at 24 h after treatment. Similar to their response to NaCl treatment, *Gmhdz51* and -*83* were also obviously upregulated by drought stress. Furthermore, the induced expression levels of *Gmhdz51* and *-83* were higher under drought treatment than under salinity treatment. By comparing the expression patterns of the 12 segmental duplicated genes, we found that three paralogous pairs, *Gmhdz2* and *-9*, *Gmhdz19* and *-27*, *Gmhdz51* and *-83*, exhibited similar expression profiles under both stress treatments. In summary, the expression patterns of the soybean HD-Zip genes detected by qRT-PCR were roughly consistent with those observed with microarray analyses, even in response to two different abiotic stress (drought and salt) treatments.

## Discussion

Preliminary analysis of HD-Zip gene family has been performed in the model plants *Arabidopsis* and rice. However, this family has not previously been studied in soybean. In the current study, we performed an overall analysis of the HD-Zip gene family in soybean, including analysis of their phylogeny, chromosomal location, gene structure, conserved motifs and expression profiles. A total of 88 full-length HD-Zip genes were identified in the soybean genome, which is 1.8 times that of *Arabidopsis*. However, our result was lower than a previous estimate, i.e., that members of the HD-Zip family are 2.9-times more abundant in soybean than in *Arabidopsis* [48]. Overall, there are two key reasons for this discrepancy. First, more and more sequences have been assembled and introduced into the soybean genome database. Second, the search methods employed in the current and previous studies differed. We used the BLASTP program (based on amino acid sequences) while the previous study used TblastN (based on nucleotide sequences). These two factors may have led to the different results obtained in the two studies.

In each subfamily, the characteristics of exon/intron structure and motif compositions were relatively conserved in recent paralogs. Indeed, previous studies have indicated that few insertions and deletions have accumulate within introns over the past 13 million years [48]. Soybean HD-Zip genes were clustered into four distinct subfamilies based on phylogenetic analysis. Compared to the *Arabidopsis*, *Populus*, rice and maize HD-Zip subfamilies, the number of soybean genes in each subfamily is much larger, implying a genome expansion of the soybean HD-Zip counterparts. The main objective of this phylogenetic study was to identify putative orthologous and paralogous HD-Zip genes. In the combined tree of the HD-Zip genes in all three species, we found only one monocot and dicot orthologous pair (*Gmhdz50* and *Os10g42490*), suggesting that the ortholog pair originated from common ancestral genes that existed before the divergence of monocots and dicots. The fact that the genes in paralogs accounted for the most of the family members confirms that soybean has undergone two duplication events after the monocot/dicot split, and most HD-Zip genes in soybean expanded in a species-specific manner.

Gene duplication is one of the major evolutionary mechanisms for generating novel genes that help organisms adapt to different environments [63], [64]. Three principal evolutionary patterns were attributed to gene duplications, including segmental duplication, tandem duplication and transposition events such as retroposition and replicative transposition [65]. Among these, segmental duplication occurs most frequently in plants because most plants are diploidized polyploids and retain numerous duplicated chromosomal blocks within their genomes [66]. In our analysis, we found that a high proportion of HD-Zip genes are distributed preferentially in duplicated blocks, suggesting that segmental duplications contribute significantly to the expansion of the soybean HD-Zip gene family. Previous studies have shown that the soybean genome has undergone two rounds of whole genome duplication, including an ancient duplication prior to the divergence of papilionoid (58–60 Mya) and a *Glycine*-specific duplication that is estimated to have occurred ∼13 Mya [48]. By calculating the duplication dates of the paralogous pairs, we demonstrated that all of the segmental duplication events in the soybean HD-Zip family occurred during the recent whole genome duplication event. The two tandem duplication pairs *Gmhdz29/-30* and *Gmhdz40/-41* were formed 9.18 and 8.95 Mya, respectively, while the segmental duplication of *Gmhdz29/-40* occurred 7.18 Mya. The results indicate that the two tandem duplication events occurred before the formation of segmental duplication.

Legumes, the third major crop worldwide, can also generate on their roots other *de novo* meristems, leading to the formation of lateral roots and symbiotic nitrogen-fixing nodules [67]. In the model legume *Medicago truncatula*, the HD-Zip I transcription factor *HB1* is expressed in primary and lateral root meristems and induced by salt stress. The mutant is also affected in nodule density (nodule number per root centimeter). In *silico* analysis, three HD-Zip I genes(*Gmhdz2, -78, -84*), three HD-Zip II genes(*Gmhdz16, -57, -77*) and one HD-ZIP III gene(*Gmhdz30*) were detected higher both in the root and noodle (Figure 5). The result indicates that these genes may affect roots and symbiotic nodules. Roots sense the edaphic water deficit, send chemical signals to shoots, and maintenance of root growth despite reduced water availability can contribute to drought tolerance through water foraging [68],[69]. The genes from HD-Zip I and II expressed broadly in the root are potentially related to regulation of developmental adaptation to environmental stress conditions such as drought. In a previous study, it was reported that *HAHB10*, a sunflower HD-Zip transcription factor belonging to subfamily II, was able to accelerate flowering and thereby shortens the plant's life cycle when ectopically expressed in *Arabidopsis* plants [70]. *Arabidopsis ATHB2/HAT4* induces early flowering when ectopically expressed and it has been described as a master switch in the shade avoidance response [33]. The tissues expression data analyses shows that 11 genes(*Gmhdz16, -21, -22, -35, -64, -70, -71, -74, -76, 77, -88*) are expressed in flowers, suggesting their putative roles in the induction of flowering. In *Arabidopsis* and rice, the function of HD-Zip III genes is related to apical embryo patterning, embryonic shoot meristem formation, organ polarity, vascular development, and meristem function [36], [71]. Leaves, the principal lateral organ of the shoot, develop as polar structures typically with distinct dorsoventrality. In comparison, the 12 soybean genes from HD-ZIP III subfamily identified in the present study showed preferentially high expression levels in young leaf, suggesting their putative roles in the regulation adaxial leaf fate. HD-ZIP IV genes have been identified in several plant species including Arabidopsis, maize, and rice, and a common feature of the vast majority of these genes resides in their epidermis-specific expression pattern [42], [72]. In maize, *OCL1* (OUTER CELL LAYER1) is expressed in the epidermis of embryo, endosperm, meristematic tissues, and organ primordia [73]. In soybean, seven genes (*Gmhdz48*, *-49*, *- 55*, *-56*, *-62*, *-68*, *-73*) from HD-ZIP IV show comparatively higher transcript abundances in seed. How these soybean HD-ZIPs perform their functional roles in the seed remains to be elucidated and further functional analyses will be required to understand their biological roles in soybean. In addition, *fwa*(flowering late) semi-dominant mutants are late flowering [74], [75], a phenotype shared by plants overexpressing *PDF*2 [20]. Two orthologous genes (*Gmhdz7,-56*) of *PDF2* were strongly expressed in the flower(Figure 5). Overexpression of *Gmhdz7* and *Gmhdz56* in soybean probably leads to a strong delay in flowering time. Soybean HD-Zip genes may also involve in other biological processes, such as fruit and seed development, for their abundant expression in the one cm pod and pod shell.

During their life cycles, the growth and productivity of plants are frequently threatened by environmental stresses such as drought and high salinity. Many stress-related genes are induced to help plants adapt to these environmental stresses. Compared with subfamily II, III, IV, subfamily I display a important role in particular in abiotic stress [11]. The Arabidopsis and rice have 17 and 14 subfamily 1 genes respectively. According to the expression patterns studied in Arabidopsis and rice, some HD-Zip family I genes are regulated by drought and salt stresses [76]–[82]. In the soybean, 8 of 23 genes were induced in both salt and drought stress and 7 genes were not presented (Fig. 4).

In this study, 12 soybean HD-Zip genes from subfamily I were predicted to be stress-related genes based on microarray data analysis, and their expression patterns under drought and salinity treatments were investigated. The results show that the 12 soybean HD-Zip genes are responsive to the two stressors. Phylogenetic analysis places *Os04g45810 (Oshox22)*, *Os02g43330 (Oshox24)*, *ATHB-7* and *ATHB-12* in the same subgroup (γ clade) of the HD-Zip family I, and they all regulate the salt and drought stress. Based on in *silico* analysis and our RT-qPCR, *Gmhdz19/-27* and *Gmhdz32/-72* belonging to γ clade also response to the two stressors. *Gmhdz51/-83* and *Gmhdz24/-38* have a close relationship with *ATHB2/-20* and *ATHB5/-6/-16* respectively. While *ATHB2/-20* and *ATHB5/-6/-16* play a role as a negative regulator under the two stress, *Gmhdz51/-83* and *Gmhdz24/-38* increased their expression level. In particular, *Gmhdz51* and *-83* were strongly induced by both stimuli. We conclude that *Gmhdz51* and *-83* may play essential roles in responses to abiotic stress. *Gmhdz2/-9* and *Gmhdz19/-27* contained to the clade ζ which is unique to the soybean are also up-regulated under the two stress. As noted above, though the closely related genes share many similar characteristics, they may have different expression patterns. Throughout evolution, they diversified and acquired new genes that may have important roles in plant development.

By comparing the six pairs of these duplicated genes, we observed that *Gmhdz2/-9*, *Gmhdz19/-27* and *Gmhdz51/-83* exhibit similar expression patterns, indicating that the responses of paralogs to stress conditions did not undergo much divergence along with the evolution of each gene after duplication and that the duplicated genes may have redundant functions in response to salinity and drought stress. By contrast, the functions of the gene pair *Gmhdz24/-38* apparently diverged under salinity treatment while those of *Gmhdz20/-72* diverged under drought treatment. The new information obtained in this study may aid in the selection of appropriate candidate genes for further functional characterization.

## Methods

### Database search and nomenclature of genes

The *Glycine max* genome database (release 1.0, http://www.phytozome.net/soybean.php) was searched to identify HD-Zip proteins using Basic Local Alignment Search Tool algorithms (BLASTP) with the published *Arabidopsis* HD-Zip protein sequences as query sequences. All obtained protein sequences were examined for the presence of the HD (PF00046, SM000389) and LZ (PF02183 SM000340) domains using the Hidden Markov Model of Pfam (http://pfam.sanger.ac.uk/search) [83] and SMART (http://smart.embl-heidelberg.de/) [84] tools. Physicochemical parameters of each gene were calculated using ExPASy (http://www.expasy.org/tools/) [85]. Information regarding cDNA sequences, genomic sequences, ORF lengths and chromosome locations was obtained from the Phytozome database.

### Phylogenetic analysis

Phylogenetic trees were constructed using MEGA 5.0 [86] with the Neighbor-Joining (NJ) method, and bootstrap analysis was conducted using 1,000 replicates with the pairwise gap deletion mode, which allows the divergent domains to contribute to the topology of the NJ tree [57]. Multiple sequence alignments of the full-length protein sequences from soybean, rice and *Arabidopsis* were also performed with MEGA 5.0 using default parameters.

### Gene structure analysis and identification of conserved motifs

Identification of the exon/intron organization of the HD-Zip genes was performed with Gene Structure Display Server (GSDS; http://gsds.cbi.pku.edu.cn/) [87] by alignment of the cDNAs with their corresponding genomic DNA sequences. Structural motif annotation was performed using the MEME program with the following parameters: number of repetitions, any; maximum number of motifs, 30 and the optimum motif widths, between six and 200 residues. In addition, structural motif annotation was performed using the Pfam (http://pfam.sanger.ac.uk/search) and SMART (http://smart.embl-heidelberg.de/) tools.

### Chromosomal location and gene duplication

The segment duplication coordinates of the target genes was detected from the SoyBase browser (http://soybase.org/gb2/gbrowse/gmax1.01/) [68]. Information about the chromosome locations was obtained from the Phytozome database. The relative duplicate blocks representing homologous chromosome segments were anchored on the 20 soybean chromosomes and indicated with the same color. Genes were considered to have undergone segmental duplication if they were found to be coparalogs that were located on duplicated chromosomal blocks, as proposed by Wei et al. (2007) [88]. Paralogs were considered to be tandem duplicated genes if the two genes were separated by five or fewer genes in a 100-kb region [89].

### Calculation of Ka/Ks values

Synonymous (Ks) and nonsynonymous substitution (Ka) rates were calculated according to a previous study [57]. Pairs from the segmental duplication events were first aligned by Clustal X2.0. Subsequently, the aligned sequences and the original cDNA sequences were analyzed by the PAL2NAL program (http://www.bork.embl.de/pal2nal/) [90], using the CODEML program of PAML [91] to estimate substitution rates. For each gene pair, the Ks value was translated into divergence time in millions of years based on a rate of 6.1×10^−9^ substitutions per site per year. The divergence time (T) was calculated as T = Ks/(2×6.1×10^−9^)×10^−6^ Mya [60].

### Microarray analysis of Gmhdzs

The genome-wide microarray data for the salt and drought stresses were downloaded from the Gene Expression Omnibus (GEO) [92] database at the National Center for Biotechnology Information under accession numbers GSE41125 (from *Glycine max*) [93] and GSE40627 (from *Glycine max*) respectively. Probe sets corresponding to the putative soybean HD-Zips were identified using an online Probe Match tool available at the NetAffx Analysis Center (http://www.affymetrix.com/). For genes with more than one probe set, the highest expression value was considered. When several genes had the same probe set, they were considered to have the same transcriptional profile.

The tissue-specific transcript data of 88 HD-Zip genes were investigated based on the RNA Seq-Atlas from fourteen tissues (http://soybase.org/soyseq/), including underground tissues (root and nodule), seed development (seed 10-DAF, seed 14-DAF, seed 21-DAF, seed 25-DAF, seed 28-DAF, seed 35-DAF and seed 42-DAF) and aerial tissues (leaf, flower, pod-shell 10-DAF, pod-shell 14-DAF and one-cm pod). The expression data were gene-wise normalized and the heatmap was drawn using Cluster (version 3.0) with the average linkage program [94].

### Plant materials, growth conditions and stress treatments

Seedlings of soybean (*Glycine max* L.) Williams 82 were used to study gene expression levels in all experiments. For expression analysis of soybean HD-Zip genes under stress, four-week-old seedlings grown in a growth chamber with a continuous 30°C temperature, a photoperiod of 12 h/12 h, 80 µmolm^−2^ s^−1^photon flux density and 50% relative humidity were used [93]. Salt stress was conducted by watering the plants with a sodium chloride (NaCl) solution at a concentration of 150 mM to saturation [95]. For drought stress, the intact roots of plants were placed onto filter paper and exposed to the air at 70–80% humidity at 25°C under dim light [57]. Seedlings without treatment were used as the control. Leaves of the stress-treated plants were collected at time intervals of 0, 1, 6, 12 and 24 h. After all of the materials were collected, they were immediately frozen in liquid nitrogen and stored at −80°C for RNA extraction. Three biological replicates were employed per sample.

### RNA extraction and qRT-PCR analysis

Total RNA was extracted from frozen samples using an RNAprep Pure Plant Kit (Tiangen) according to the manufacturer's instructions. The first-strand cDNA was then synthesized using a PrimeScriptTM RT Reagent Kit (TaKaRa). Gene-specific primers were designed using Primer5.0, and their specificity was checked using information provided on the NCBI website. CYP2 (cyclophilin), a constitutively expressed soybean housekeeping gene, was used as reference for normalization [96]. RT-PCR was performed in a 25 µl volume containing 12.5 µl 2×SYBR® Premix Ex Taq™ (TaKaRa), 1 µl diluted cDNA, 0.15 µl of each gene-specific primer and 11.2 µl ddH_2_O. The PCR conditions were as follows: 95°C for 10 min, 40 cycles of 15 s at 95°C, 58°C for 1 min. Three biological replicates were used per sample. The relative expression level was calculated as 2^−ΔΔCT^ [ΔC_T_ = C_T, Target_−C_T, CYP2_. ΔΔC_T_ = ΔC_T, treatment_−ΔC_T, CK (0 h)_].The relative expression level (2^−ΔΔCT, CK (0 h)^) in the control plants without treatment was normalized to 1 as described previously [97]. Statistical analyses were performed using SDS software 1.3.1 (Applied Biosystems).

## Supporting Information

Text S1 **The information of 88 Gmhdz genes.** A complete list of 88 HD-ZIP gene sequences identified in the present study. Genomic DNA sequences are obtained from Phytozome (http://www.phytozome.net/soybean, release 1.0). Amino acid sequences are deduced from the corresponding coding sequences.
(TXT)

Figure S1 **Motifs of 88 soybean HD-Zip proteins.** Thirty motifs were identified through MEME (http://meme.nbcr.net/meme/), and then motif organizations of 88 soybean HD-Zips were investigated through MAST (http://meme.nbcr.net/meme/).
(TIF)

Table S1 **The detailed information on conserved amino acid sequences and lengths of the 30 motifs.**
(XLSX)

Table S2 **Recent Synteny blocks of soybean and soybean (13 Mya) genomes containing HD-ZIP genes.**
(XLS)

Table S3 **Pairwise identities between paralogous pairs of HD-ZIP genes from Soybean.**
(XLS)

Table S4 **The probes and microarray data of soybean HD-ZIP genes.**
(XLS)

Table S5 **The transcriptions of soybean HD-Zip genes through RNA-seq analysis.**
(XLS)

Table S6 **A list of primer sequences of the 12 selected HD-ZIP genes for qRT-PCR analysis.**
(XLS)
