# Supplementary figures and images for: Genome-Wide Analysis of Soybean HD-Zip Gene Family and Expression Profiling under Salinity and Drought Treatments
Source: PLoS One. 2014 Feb 3;9(2):e87156. doi: 10.1371/journal.pone.0087156 (PMC3911943; doi:10.1371/journal.pone.0087156)

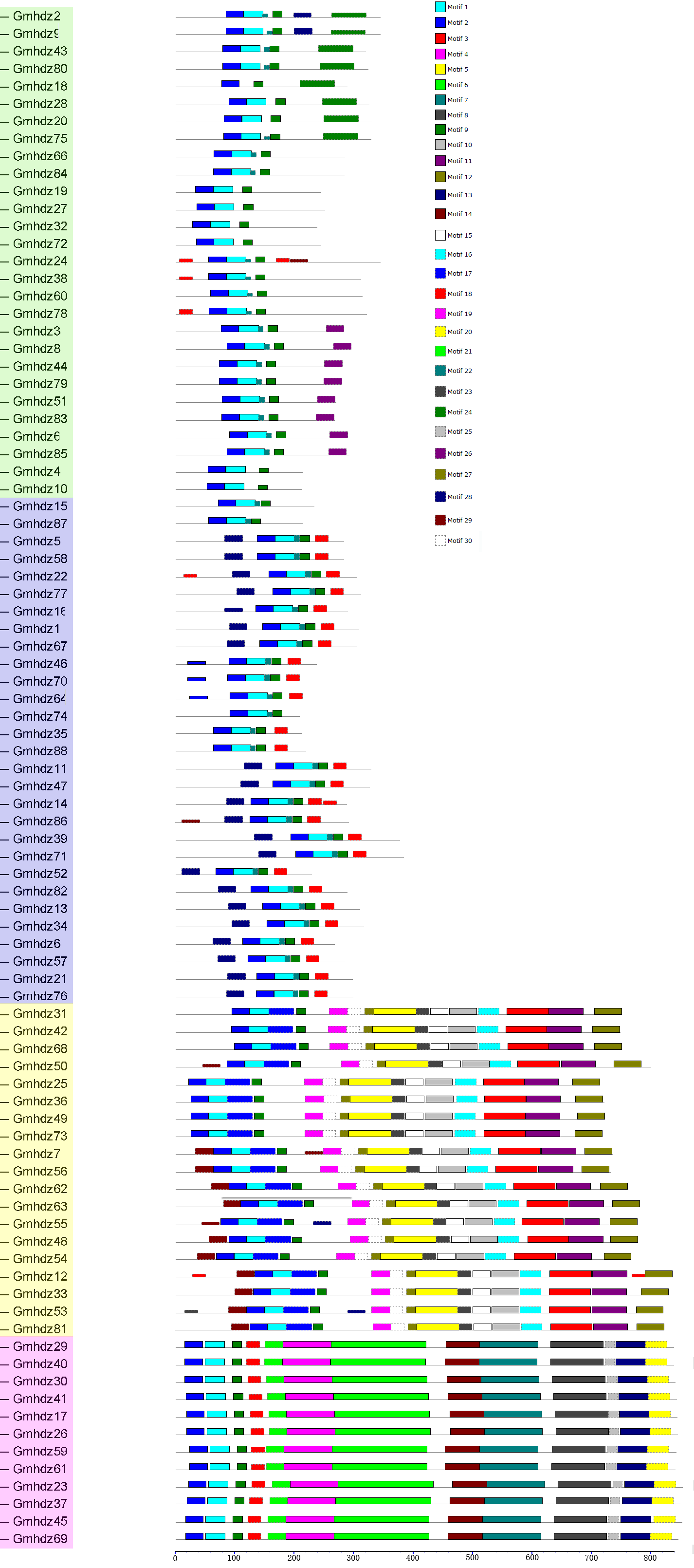

Supplement: Figure S1 — Motifs of 88 soybean HD-Zip proteins. Thirty motifs were identified through MEME (http://meme.nbcr.net/meme/), and then motif organizations of 88 soybean HD-Zips were investigated through MAST (http://meme.nbcr.net/meme/). (TIF) [file pone.0087156.s002.tif]
